# Notes and News

**Published:** 1897-06-12

**Authors:** 


### NOTES AND NEWS.

(Collected and Communicated.)

HOSPITAL MEETINGS.

North-Eastern Hospital for Children, Hackney Road.—Annual Meeting at the Hospital, Thursday, June 17th, 4 p.m.

The Prince of Wales has further shown his appreciation of the work of the Royal London Ophthalmic Hospital by a donation of ten guineas to the institution.

Mr. John Temple, of Aigburth, Liverpool, has endowed a bed at the Hahnemann Hospital in that city. The bed is to bear Mr. Temple's name.

The treasurer of Guy's Hospital has received £10,000 from the trustees of the late Mr. William Andrew Guesdon towards the completion of Guy's Re-endowment Fund, which now stands at £194,000.

The hospitals are not behindhand in tending their congratulations to Her Majesty on the completion of her long reign, and most of them have already sent congratulatory addresses.

The Secretary of the Great Northern Central Hospital draws attention to the danger which attends the receipt of postal orders, representing small donations and subscriptions, when not properly safeguarded by being crossed to the order of the hospital.

The treasurer of Charing Cross Hospital has received from the executors of the late Mr. Andrew Guesdon a cheque for £3,000, being the share assigned to the hospital by the executors from the residue of Mr. Guesdon's estate, bequeathed by him for distribution amongst charities. This amount has been placed to the credit of the Special Appeal Fund, which has now reached the sum of £30,749. A sum of £1,000 has also been received from Dowager Lady Loder to endow a bed in memory of her husband, the late Sir R. Loder, M.P.

A proposal has been made to decorate the base of the Albert Memorial on Jubilee Day with flowers, growing and otherwise, and to distribute the former amongst the hospitals afterwards. There can be no doubt that this project, if carried out, will be very pleasing to the Queen. Growing plants are always especially welcomed in hospital wards, and we learn that many welcome gifts of this kind were made to the hospitals after the Duchess of Albany's bazaar at the Imperial Institute.

The distribution of prizes to the students of the Charing Cross Hospital Medical School took place last week in the theatre of the Institution, Field Marshal Sir John Lintorn Simmons presiding.

The appointment of Professor of Clinical and Military Medicine in the Army Medical School at Netley has been bestowed upon Brigade-Surgeon Lieut.-Colonel Kenneth McLeod, M.D., LL.D., F.R.C.S.Edinburgh.

The Croonian Lectures of the Royal College of Physicians will be delivered by Dr. Hale White on Tuesdays and Thursdays, at five p.m., June 15th, 17th, 24th, an 29th, the subject being "The Means by which the Temperature of the Body is Maintained in Health and Disease."

Quite a novel means of securing funds for a hospital has been devised on behalf of the Mater Misericordiæ Hospital, in Belfast. A trip to Scotland was arranged last Monday, in a large steamship capable of carrying 1,500 passengers. There was a large demand for tickets, and a substantial sum will probably be handed over to the hospital.

A very interesting exhibition of a working coal mine is now to be seen at the Crystal Palace, it having been opened at the Queen's Birthday fête there on May 26th in aid of the Prince of Wales's Hospital Fund. The mine has been constructed under the floor of the great nave, and is a wonderfully realistic illustration of the conditions under which coal is obtained by means of the latest appliances.

The Hospital Saturday Collection at Birmingham is now progressing. Special efforts are being made to raise the sum to £20,000 this year in commemoration of the Queen's long reign. On Saturday upwards of £12,500 was contributed, being a sum considerably in excess of the first day's collection last year; so it is to be hoped that even if the £20,000 is not obtained, at any rate last year's collection of £15,000 will be exceeded.

The Lord Mayor of York has recently laid the foundation-stone of the new dispensary for York. The old buildings are now inadequate for the purpose. A good site upon which a building worthy of the city could be erected was first acquired, and the £2,000 required to erect the institution has been in part provided for through the Rawdon bequest, and will be further supplemented by the Jubilee collection in the city. The building will be of three storeys, with a handsome frontage of 73 feet. It is to be fitted with all modern appliances.

A new wing at the Samaritan Hospital, Belfast, has been opened by Mrs. Forster Green and Miss Benn, both of whom are associated in many good services on behalf of the hospital. Towards the present addition Mr. Forster Green contributed £500. The new wing contains two wards, in separate storeys, each containing four beds. Lavatories, separate offices, and nurses' quarters are provided, as these wards are intended for isolation cases. The readjustment rendered possible by the addition of the new wing has permitted of many improvements in the older part of the institution to be carried out.

